# Extraordinary heat insulation in RbAg_4_I_5_

**DOI:** 10.1093/nsr/nwag318

**Published:** 2026-05-30

**Authors:** Ziyue Liu, Qingyu Bai, Zhiwei Chen, Linjie Wu, Te Kang, Changyuan Li, Long Yang, Jun Luo, Yanzhong Pei

**Affiliations:** Interdisciplinary Materials Research Center, School of Materials Science and Engineering, Tongji University, Shanghai 201804, China; Interdisciplinary Materials Research Center, School of Materials Science and Engineering, Tongji University, Shanghai 201804, China; Interdisciplinary Materials Research Center, School of Materials Science and Engineering, Tongji University, Shanghai 201804, China; Interdisciplinary Materials Research Center, School of Materials Science and Engineering, Tongji University, Shanghai 201804, China; Interdisciplinary Materials Research Center, School of Materials Science and Engineering, Tongji University, Shanghai 201804, China; Interdisciplinary Materials Research Center, School of Materials Science and Engineering, Tongji University, Shanghai 201804, China; Interdisciplinary Materials Research Center, School of Materials Science and Engineering, Tongji University, Shanghai 201804, China; Interdisciplinary Materials Research Center, School of Materials Science and Engineering, Tongji University, Shanghai 201804, China; Interdisciplinary Materials Research Center, School of Materials Science and Engineering, Tongji University, Shanghai 201804, China

**Keywords:** thermal conductivity, superionic conductor, porous heat insulator, thermoelectric device

## Abstract

Superionic conductors are characterized by highly mobile charged ions, reflecting the presence of loosely bonded and partially occupied species within their crystal structures. These structural features are also essential for achieving high-performance thermal insulation. However, conventional skeleton materials used for thermal insulation, such as SiO_2_, lack such crystallographic attributes. As a result, room-temperature thermal conductivity (*κ*) values for fully dense and 3%-dense SiO_2_ remain around 1400 and 15 mW m^−1^ K^−1^, respectively. This work demonstrates the use of superionic conductors as a new paradigm for thermal insulation. Single-crystalline RbAg_4_I_5_ is shown to exhibit an exceptionally low *κ* of 130 mW m^−1^ K^−1^ at room temperature. Furthermore, a 3.6%-dense porous form of RbAg_4_I_5_ achieves a record-low *κ* of only 6 mW m^−1^ K^−1^, substantially outperforming existing porous thermal insulators. When applied as filler in a conventional Bi_2_Te_3_ thermoelectric device, this porous insulator suppresses gas-mediated heat transfer, leading to a 10% improvement in device performance. In addition, this material effectively isolates heat from a CPU to enable a temperature drop of 5.3 K for the adjacent flash memory, thus achieving a 16% improvement in its startup speed. The design strategy introduced here is expected to open new pathways for advancing thermal management technologies.

## Teaser

Superionics serve as thermal insulation skeletons, making porous RbAg₄I₅ delivers superior heat insulation far beyond air.

## INTRODUCTION

Heat insulators, which resist heat flow across a temperature gradient, become crucial components in modern technologies including thermal barrier coatings [1], thermal managements [2] and thermoelectrics [3]. To explore the fundamental mechanisms of heat insulation in solids, a variety of materials from natural wood [4] to artificial materials [5,6] have been investigated. In these studies, the thermal conductivity (*κ*) is a key property indicator.

Heat flow is mediated by the transport of particles and quasi-particles. For practical applications requiring mechanical robustness, heat insulators are typically solids. Heat conduction can stem from diffusible phonons/magnons/electrons [7–9] and movable molecules/ions [10], e.g. the heat in the form of radiative photons can traverse from hot side to cold side in both vacuum and media. Therefore, the essence for heat insulation lies in effectively suppressing the transport of these energy carriers.

Since heat transport channels of diverse carriers collectively contribute to total heat conduction in parallel, the thermal conductivity is primarily determined by the large-flux channels. For example, heat radiation via photons is negligible in non-transparent SiO_2_, where the *κ* of 1400 mW m^−1^ K^−1^ stems from the transport of phonons [11]. Whereas gas molecules encapsulated in highly porous SiO_2_ substantially elevate their contribution to heat conduction, since the minimal use of SiO_2_ skeleton produces a huge thermal resistance by impeding phonon transports [12].

Current efforts on enhancing heat insulation are focused primarily on the manipulation of porous structures inside the skeleton [13,14] and pore-stuffing [15]. At low relative densities, the aerogel thermal conductivity is dominated by the porous network structure [16], inter-particle contact resistance [17], gas-phase Knudsen effect [18] and fabrication conditions [19], rather than the intrinsic thermal conductivity of the solid skeleton. Alternatively, when the solid skeleton becomes percolated and contact effects are less severe, the contribution of the solid skeleton to the low thermal conductivity gradually becomes important and cannot be neglected at sufficiently high relative densities.

SiO_2_ aerogels are typical commercial heat insulators, and their dense nano-scale pores significantly impede the flow of encapsulated gas molecules. Although the transport of hot gases is successfully minimized by such a porous structure, a further reduction in heat transport via lattice vibrations of the solid skeleton is crucial for decreasing the heat conduction of the whole insulator [20,21]. Existing solid skeletons of aerogels are thermally stable, while these materials are insufficiently heat-insulative. Examples include graphene [22,23], 2D-BN [24] and SiO_2_ [11] with thermal conductivities of 2000, 400 and 1.4 W m^−1^ K^−1^, respectively. It is then straightforward that a switch to skeleton materials of better heat insulation would fundamentally offer further advancements for porous heat insulators.

Solid-state superionic conductors inherently possess highly mobile ions, scientifically indicating the existence of loosely linked bonds with partially occupied atomic sites in the lattice structure. Essentially, the interatomic bonding force [25], the mass of bonded atoms [26] and the concentration of phonon scattering centers [27] determine the lattice vibrational properties and thus the phononic heat conduction [28]. This directs our focus to the RbAg_4_I_5_ superionic conductor of electrical insulation [29] and heavy average atomic mass (115.16 g mol^−1^, one of the heaviest among superionics) as a heat insulator candidate. Meanwhile, porosification is also a common strategy to reduce the thermal conductivity of composites. Introducing porosity into inorganic materials with low thermal conductivity is a widely applicable approach [13], which can also be extended to superionic conductors.

This study demonstrates that single-crystalline RbAg_4_I_5_ superionic conductor inherently exhibits a low *κ* of only 130 mW m^−1^ K^−1^ at 300 K, owing to its weak bonds, dense lattice vacancies and large primitive cell [30,31]. Few superionic conductors have been reported to show total thermal conductivities down to 200 mW m^−1^ K^−1^ [32,33] because the presence of conducting electrons in those materials contributes substantially to the heat conduction [34,35]. To date, there are also rare reports of dense solids possessing a *κ* of less than 200 mW m^−1^ K^−1^ at room temperature [36–39]. Moreover, RbAg_4_I_5_ can be processed with an organic solvent using a dry-freezing technique, achieving a porosity of up to 96.4% with nano-scale pores inside the skeleton. This eventually realizes a room-temperature *κ* of only 6 mW m^−1^ K^−1^ in 3.6% dense RbAg_4_I_5_, outperforming known porous heat insulators.

## RESULTS AND DISCUSSION

**Figure 1.**
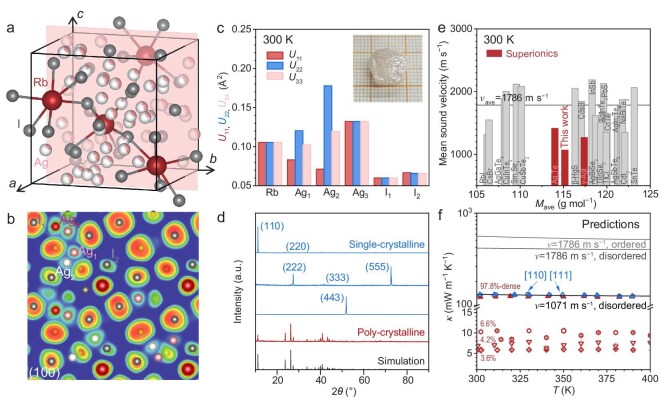
(a) The *P*4_1_32 lattice structure of RbAg_4_I_5_ with partial occupation of Ag ions. (b) Room-temperature electron-density mapping of the (100) plane. (c) Anisotropic atomic displacement parameters (*U*_11_/*U*_22_/*U*_33_) of Rb, Ag and I atoms, indicating the weak Ag-I bonds. (d) X-ray diffraction patterns for polycrystalline and single-crystalline RbAg_4_I_5_. (e) Mean sound velocity for RbAg_4_I_5_, as compared to that of materials with a similar average atomic mass (more details and the relevant references are given in Table S4). (f) Temperature-dependent thermal conductivity for single-crystalline and polycrystalline RbAg_4_I_5_, along with the predictions with and without the effect of Ag disorder.

Focusing on the skeleton of porous heat insulators, superionic RbAg_4_I_5_ has been selected as the typical example. The synthesis procedures of both single- and poly-crystalline samples are illustrated in Fig. S1. The *P*4_1_32 structure is identified by single-crystal X-ray diffraction analysis (Fig. 1a and Table S2), where Ag^+^ ions are found to be highly disordered with partial occupancies at all three distinct sites [40]. The increase in lattice parameter with increasing temperature corresponds to a thermal expansion coefficient of 25 × 10^−6^ K^−1^ (Fig. S2). The phase purity and homogeneity of single crystalline RbAg_4_I_5_ are verified by the scanning electron microscope (SEM) equipped with an energy dispersive spectrometer (Fig. S2d). Room-temperature electron-density mapping of (100) plane reveals the existence of isolating Ag ions (Fig. 1b), indicating the weak Ag-I bonds. The overall weakness of bond strength and the existence of vacant sites contribute to very large atomic displacement parameters (ADP, *U*_11_/*U*_22_/*U*_33_) of Ag (Fig. 1c).

The as-grown crystal is sliced along a few specific crystallographic directions (Fig. 1d). Most importantly, the existence of weak chemical bonds would lead to a lattice softening for a reduction in mean sound velocity (*v*). To further rule out the effect of atomic mass on *v*, various superionic conductors with comparable average atomic mass of 105–125 g mol^−1^ are collected for comparison. These superionic materials demonstrate low sound velocity, with RbAg_4_I_5_ exhibiting the lowest sound velocity and thermal conductivity among them (Fig. 1e). This consistently indicates that superionic behavior has a strong effect on reducing *v* (from an average of 1786 to 1071 m s^−1^ in RbAg_4_I_5_, as given in Table S3).

Due to the cubic lattice structure, the measured thermal conductivities along the [110] and [111] directions are nearly isotropic, with values as low as 130 mW m^−1^ K^−1^ within 300–400 K range (Fig. 1f). Poly-crystalline RbAg_4_I_5_ shows a slightly reduced thermal conductivity of ∼125 mW m^−1^ K^−1^ (Fig. S3), which might be attributed to the enhanced phonon scattering at grain boundaries. The existence of highly disordered Ag^+^ ions (Fig. S4) and a large amount of lattice vacancies would lead to strong phonon scattering as well. According to the point-defect scattering model based on Boltzmann transport theory taking into account lattice periodicity [41,42], hypothetical RbAg_4_I_5_ without partial-occupancy induced disordering (assuming *v* = 1786 m s^−1^), is predicted to show a *κ* of 417.6 mW m^−1^ K^−1^ (Fig. 1f), which is about triple of the measurement in this work. The modeling suggests the superionic behavior in RbAg_4_I_5_ not only reduces the propagating velocity but also strengthens the scattering of phonons, which eventually leads to its extremely low thermal conductivity, which is highly advantageous in the skeletons of porous heat insulators (Fig. S5 and Table S5). It is worth noting that the above analysis of phonons is only applicable to rigid lattice frameworks and is used to explain the intrinsic low thermal conductivity caused by complex unit cells, heavy elements and strong phonon scattering [43,44].

The thermal conductivity of RbAg_4_I_5_ is nearly constant in the range of 300–400 K. This might suggest that the temperature trend of thermal conductivity is almost determined by lattice vibrations, and the contribution of ionic enthalpy diffusion is negligible within the experimental error. This is because the contribution of ionic enthalpy diffusion to thermal conductivity necessarily increases monotonically with increasing temperature. Meanwhile, ionic conductivity is highly density-dependent, which decreases significantly with increasing porosity [45]. The mechanism for the observed lower thermal conductivity in porous materials will be discussed later in detail.

**Figure 2.**
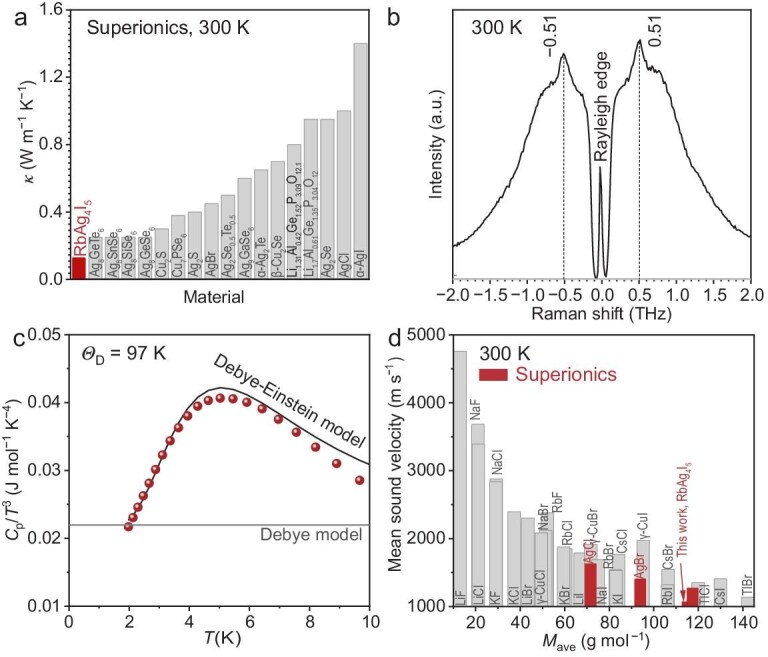
(a) Room-temperature thermal conductivity for polycrystalline RbAg_4_I_5_ compared with other superionic conductors (more details and the relevant references are given in Fig. S6). (b) Raman spectrum for RbAg_4_I_5_ at room temperature. (c) Temperature-dependent heat capacity, along with the predictions according to the Debye and Debye–Einstein models. (d) Average-atomic-mass-dependent mean sound velocity for halides at 300 K.

Compared with other superionic conductors, RbAg_4_I_5_ contains relatively heavy elemental components. As can be clearly seen from Fig. S6, the average atomic mass of RbAg_4_I_5_ is much larger than that of other superionic conductors such as Cu_2_Se, making the thermal conductivity (*κ*) of RbAg_4_I_5_ much lower (Fig. 2a). A Raman-active optical phonon mode with an extremely low frequency (∼0.5 THz) is observed as shown in Fig. 2b. At room temperature and above, the specific heat capacity (*C*_p_) measured at a constant pressure is comparable to that predicted by the Dulong-Petit law (Fig. S7). To maintain consistency in the thermal conductivity estimation with literature reports, the use of the Dulong-Petit limit in this work is reasonable and conservative in the temperature range of 200–400 K [40,46]. At low temperatures, *C*_p_ deviates from the predicted *T*^3^ relationship based on the Debye model, due to the existence of low-frequency optical phonon modes. Once these low-frequency optical modes are occupied by the thermally activated phonons, they would significantly contribute to the heat capacity beyond the Debye prediction with a Debye temperature (*Θ*_D_) of 97 K (Fig. 2c). Including one more Einstein mode with an Einstein temperature (*Θ*_E_) of 25 K, the *C*_p_/*T*^3^ can be well reproduced. The *Θ*_E_ of 25 K corresponds to the frequency of 0.52 THz contributed from the optical modes, which quantitatively agrees well with the observed Raman-active optical phonon modes. This is further consistent with the quite similar disordering and partial occupancy characteristics in the low-temperature phases [40,47].

A survey of average atomic mass (*M*) dependent mean sound velocity (*v*) for halides (Fig. 2d) clearly suggests a reduction in *v* in ionic conductors, which can be qualitatively understood by the weak chemical bonds that enable ions to be highly mobile. Meanwhile, RbAg_4_I_5_ with a more complex unit cell structure and the presence of partial occupancies of atoms further modulate chemical bonding strength. Thereby, a sound velocity of 1056 m s^−1^ lower than that of other halide-based superionic conductors is obtained in RbAg_4_I_5_.

**Figure 3.**
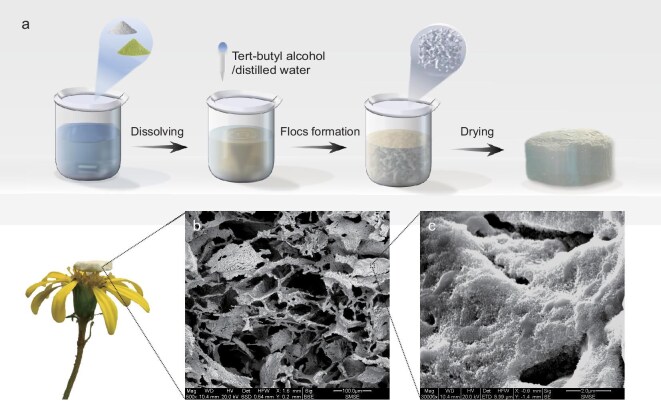
(a) Schematic for the fabrication of porous RbAg_4_I_5_. Scanning electron microscopy images taken at low (b) and high (c) magnifications for the 3.6%-dense sample.

Highly porous RbAg_4_I_5_ can be synthesized by a solution-processed method. In this work, dimethyl sulfoxide (DMSO) is used as the solvent to dissolve the mixture of a 1:4 molar ratio of RbI and AgI raw powders. As compared to commonly used *N, N*-Dimethylformamide [48] organic solvent for inorganic salts, DMSO [49] has a higher boiling point (189°C), lower saturated vapor pressure (76.60 kPa at 0°C), lower volatility and higher coordination affinity, which are beneficial for the formation of porous structures [50]. With the addition of tert-butyl alcohol and distilled water, RbAg_4_I_5_ flocs form a cross-linked network structure. The density of the sample can be controlled by adjusting the evaporation speed of organics during the dry-freezing procedure (Fig. 3a). In this way, porosity up to 96.4% can be achieved. On the other hand, the densities of 60% or above can be obtained by controlling the profile of hot pressing polycrystalline RbAg_4_I_5_ powders (Fig. S1). This process of fabrication confirms that the target porous microstructures are reproducible and controllable, this approach can also be extended to introduce porosity into other superionic conductors. The phase purity of solution-processed porous RbAg_4_I_5_ is confirmed by the X-ray photoelectron spectroscopy and X-ray diffraction patterns (Fig. S8).

The structural investigation provides the side length of the cavity (*d*), which is a key variable for determining the gas thermal conductivity (*κ*_g_). We quantified the porous structural parameters by surface area measurements of Brunauer−Emmett−Teller revealing a surface area as large as 210 m^2^ g^−1^ (Fig. S9a), suggesting that the mesoporous structure is mainly concentrated at a diameter of ∼45 nm (Fig. S9b). And SEM images reveal a hierarchical honeycomb-like structure in the 3.6% dense RbAg_4_I_5_ sample (Fig. 3b), where the large-sized pores are about tens of micrometers in average diameter. Importantly, the wall of honeycomb consists of numerous inter-linked nano-sized bubbles (Fig. 3c). Since the existence of mobile Ag^+^ in RbAg_4_I_5_ challenges the stability under electron beams, higher-magnification SEM images in this work are unavailable. Such a hierarchical microstructure would significantly limit the mean free path of gases encapsulated in the nano-scale pores [51,52], leading the heat transport of confined gases to be lower than that of free ones [20,53]. These structural investigations validate the reliability of the observed low thermal conductivity.

**Figure 4.**
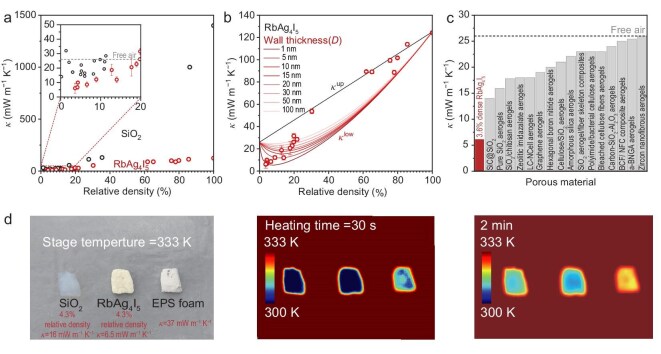
(a) Room-temperature thermal conductivity as a function of density for porous RbAg_4_I_5_, SiO_2_ and SiO_2_-based heat insulators. (b) Comparison between measurements and model predictions for RbAg_4_I_5_ at 300 K. (c) A comparison of room-temperature thermal conductivity among the porous materials in this work and literature (more details and the relevant references are given in Table S6). (d) A comparison of heat insulation between RbAg_4_I_5_, commercial SiO_2_ aerogel and EPS foam of similar density placed on a hot stage of 333 K, monitored by an infrared camera for different heating times.

The measured thermal conductivity (*κ*) of porous RbAg_4_I_5_, consisting of the contributions from both solid and gas phases, and the measured mean sound velocity (*v*) are found to continuously decrease with the decrease in density (Figs 4a and S10). As compared to existing SiO_2_-based materials, porous RbAg_4_I_5_ in this work shows significantly lower *κ*, which can be understood by its much lower intrinsic *κ* (125 mW m^−1^ K^−1^ versus 1400 mW m^−1^ K^−1^ in dense form).

A model [54] of uniformly distributed cubic pore structure is developed to understand the thermal conductivity of porous materials (Fig. S11), which takes into account porosity-induced reduction in sound velocity of skeleton [12,55] and in molecule transport due to gas confinement (pore size of mean free path of free air or smaller) [20,53]. Meanwhile the interfacial thermal resistance is neglected for intrinsic phonon scattering plays a key contributory role in the solid thermal conductivity at high relative densities (Fig. S12). This model offers effective guidance for designing the hierarchical structure for minimizing *κ* (Fig. 3c). It is worth noting that different atmospheres affect the thermal conductivity as well, which mainly acts on the gas thermal conductivity component of porous materials. The experimental thermal conductivity and the modeling predictions under different atmospheres are shown in Fig. S13. As expected, the porous RbAg_4_I_5_ exhibits the lowest thermal conductivity under vacuum conditions, due to the reduced contribution of gases to thermal conductivity. This model under air suggests a *κ* of 6 mW m^−1^ K^−1^ (Fig. 4b) in 3.6%-dense porous RbAg_4_I_5_ of *d* = 45 nm (Fig. S9b), which is quite consistent with the model predicted minimum of 5.4 mW m^−1^ K^−1^. The estimated error bars in thermal conductivity are based on the three to five times repeated measurements by Laser flash method, and the raw data of thermal diffusivity measurement for a series of porous samples at 300 K shown in Fig. S14 [56,57].

The stability of porous material against moisture is a key property for practical uses [58]. The anionic framework of RbAg_4_I_5_ consists of I^−^ and metal cations (Ag^+^, Rb^+^), which exhibit extremely weak hydrogen-bonding interactions with water molecules. Moreover, the material surface lacks strong polar sites such as hydroxyl groups and unsaturated bonds, so water molecules can only attach to the pore surfaces via physical adsorption and cannot penetrate the lattice. In our experiments, the matrix material of RbAg_4_I_5_ shows a water solubility of only 0.133 g/100 mL, indicating that it is nearly insoluble in water (Table S6). To minimize the potential influence of moisture absorption on the transport property measurements, all property measurements of the porous materials in this study were carried out immediately after sample preparation. The thermal conductivity of 3.6% dense RbAg_4_I_5_ here is markedly lower (Fig. 4c and Table S7), compared to porous materials form the literatures [13,52,59–64]. As shown in Fig. 4d and Fig. S15, all materials are placed on a heat stage of 333 K. The much hotter upper surface of 4.3%-dense SiO_2_ aerogel (*κ* = 16 mW m^−1^ K^−1^) and EPS foam (*κ* = 37 mW m^−1^ K^−1^) observed after 2 min, suggests its inferior heat insulation to that of 4.3%-dense RbAg_4_I_5_ (*κ* = 6.5 mW m^−1^ K^−1^) developed in this work.

**Figure 5.**
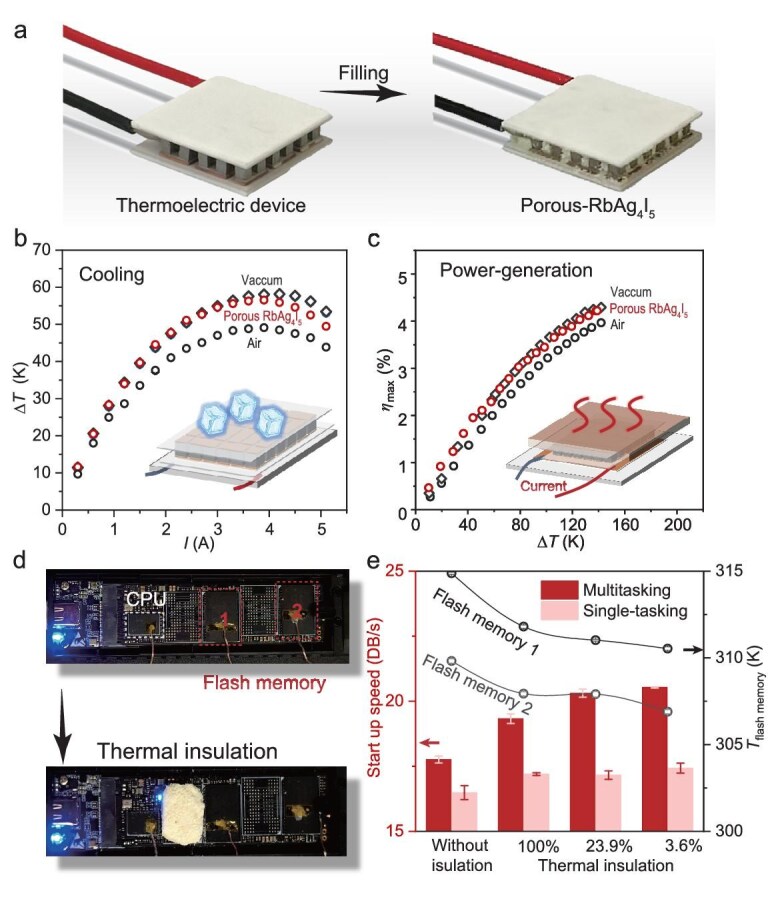
(a) Thermoelectric devices with and without filling of ∼4%-dense porous RbAg_4_I_5_. (b) Measured maximum cooling temperature drop and (c) the conversion efficiency (*η*_max_) of thermoelectric devices before and after filling. (d) Schematic diagram of porous material isolating heat conduction from the CPU. (e) Temperature change curve and hard drive startup speed during operation with and without CPU thermal insulation conditions.

To demonstrate the extraordinary heat insulation in practical applications, ∼4%-dense porous RbAg_4_I_5_ was filled into a commercial Bi_2_Te_3_ thermoelectric device (Model 9501/017/030 B from Ferrotec) (Fig. 5a, Fig. S16 and Table S8) to minimize the heat leakage under a temperature gradient. In ambient air and at room temperature, a maximum cooling temperature difference (Δ*T*_max_) of 56.5 K is achieved in the RbAg_4_I_5_-filled device, which is 7.6 K higher than that in the unfilled device. The Δ*T*_max_ of 56.5 K closely approximates the performance of the unfilled device in vacuum (Fig. 5b). This again indicates the thermal conductivity of 3.6%-dense porous RbAg_4_I_5_ is much lower than that of free gases. For thermoelectric power-generation with a room-temperature cold side, the conversion efficiency (*η*_max_) increases by 10% compared to the literature report [35] due to the filling of porous RbAg_4_I_5_ (Fig. S17). Furthermore, this porous material effectively isolates heat conduction from CPU, leading to a temperature drop of 5.3 K in the adjacent flash memory and a corresponding 16% improvement in its startup speed (Fig. 5d and e). A load-bearing capacity was carried out for this sample, demonstrating that the synthesized porous RbAg_4_I_5_ possesses a certain degree of structural integrity under low-stress and static loading conditions (Fig. S18). Fiber reinforcement or polymer cross-linking strategies [65] will further improve the toughness and mechanical strength, which will give it a broader scope of application.

## CONCLUSION

Taking the features of weakly bonded ions and partial atomic occupancy in superionic conductors, single-crystalline superionic RbAg_4_I_5_ shows a remarkably low thermal conductivity of 130 mW m^−1^ K^−1^ at room temperature. By further manipulating its hierarchical structure of pores through a solution processing, the 96.4%-porosity RbAg_4_I_5_ material successfully realizes a record low thermal conductivity of 6 mW m^−1^ K^−1^ at room temperature. This work demonstrates that superionic conductors with mobile ions have great potential as superior solid skeletons for porous heat insulators and thermoelectric conversion.

## Supplementary Material

nwag318_Supplemental_File
