# Supplementary material for: Extraordinary heat insulation in RbAg4I5
Source: Natl Sci Rev. 2026 May 30;13(13):nwag318. doi: 10.1093/nsr/nwag318 (PMC13348246; doi:10.1093/nsr/nwag318)
Supplement: nwag318_Supplemental_File [file nwag318_supplemental_file.pdf]

Supplementary

Extraordinary heat insulation in $RbAg_4I_5$

Ziyue Liu#, Qingyu Bai#, Zhiwei Chen*, Linjie Wu, Te Kang, Changyuan Li, Long Yang, Jun Luo* and Yanzhong Pei*

Interdisciplinary Materials Research Center, School of Materials Science and Engineering, Tongji Univ., 4800 Caoan Rd., Shanghai, 201804, China

*Email: 14czw@tongji.edu.cn (ZC), junluo@tongji.edu.cn (JL), yanzhong@tongji.edu.cn (YP)

Materials and Methods

Synthesis: High-purity RbI (99.9%, Aladdin) and AgI (99.99%, Aladdin) powders were loaded and sealed in the quartz ampoules under vacuum. The ampoule was heated up to 973 K and held for 2 hours, followed by cooling to 673 K and quenched into cold water. The product was annealed at 423 K for 48 hours. To obtain pellet samples with 60% to 97% of the theoretical density, the resulting ingot was hand-ground into fine powders for hot pressing under various conditions: temperature of 393 to 408 K, duration times of 10 to 30 minutes, and uniaxial pressures of 1 to 45 MPa. The single crystalline $RbAg_4I_5$ was grown using a vertical temperature gradient freeze technique at 493 K with a cooling rate of 0.7 K/h.

Phase characterization: Phase identification and structural analysis of $RbAg_4I_5$ were characterized by X-ray diffraction (DX2000, Cu-Kα radiation; Rigaku SmartLab, Cu-Kα radiation; Bruker D8 VENTURE diffractometer with a PHOTON II CCD detector, Mo-Kα radiation). The single crystal structure was then resolved and refined by SHELXT and OLEX2(*1, 2*). The microstructure and chemical composition of the single crystalline $RbAg_4I_5$ were characterized by scanning electronic microscope (SEM, Phenom Pro) equipped with an energy dispersive spectrometer (EDS). The phase composition of the porous $RbAg_4I_5$ samples was analyzed by X-ray electron spectroscopy (XPS, Al-Kα radiation, Thermo Fisher Scientific).

Property measurements: The optical band gap was measured by UV-3600 Plus (Shimadzu). The ionic conductivity was carried out by Chenhua 760 electrochemical workstation. The thermal conductivity (κ) is estimated by $\kappa=\rho^*C_p\lambda$, where ρ^* is the density measured by mass-volume method. In details, a vernier caliper (precision: ±0.01 mm) was used to measure the diameter (R) and thickness (t) of samples, where the relative errors were 0.2% for diameter and 5% for thickness. The sample mass was measured using an electronic balance (Ohaus PX224ZH/E, precision: ±0.1 mg) with a relative error of 0.2%. The density was calculated as $\rho^*=m/(\pi[R/2]^2t)$, and its relative error was less than 0.3%. C_p is the heat capacity (using the Dulong-Petit limit of heat capacity in this work), λ is the thermal diffusivity measured by the laser flash method (Netzsch LFA467). All samples were around 1 mm thick and tested in an air atmosphere. For porous samples, the absorption of pulse energy is no longer limited to the front surface but extends to a thin layer along the sample thickness. Studies have shown that this results in an exponential decay of the initial temperature distribution within the sample(*3*). Therefore, the Standard model for dense materials is no longer applicable for fitting the thermal diffusivity curve. The Penetration model is used in this work, which introduces a penetration depth as an exponential term (a fitting parameter describing the extent of energy penetration into the material, also known as the mean free path of photons). Because the samples were synthesized with different porosities in this work, the Standard model or Transparent model and Penetration model were used to fit the thermal diffusivity for the dense and porous samples, respectively. The thermal diffusivity of each sample was measured repeatedly by 3-5. The uncertainty of the final model fitting for thermal diffusivity was less than 2% (ranging from 0.2% to 1.6%), indicating an excellent match between the model predictions and experimental data. The heat capacity was measured by a physical properties measurement system (PPMS, Quantum Design). The sound velocity was measured at room temperature using an ultrasonic pulse-receiver (Olympus-NDT) equipped with an oscilloscope (Keysight). Raman spectroscopy was carried out by LABRAM HR-EVOLUTION Raman spectrometer (HORIBA) with excitation laser of 532 nm, equipped with an ultra-low frequency filter to observe the low frequency Raman active modes. Infrared photos were taken by HINMICRO K20.

DFT calculation method: The first-principle calculation was performed with VASP code (*4, 5*) using Perdew-Burke-Ernzerhof (PBE) exchange-correlation function (*6*), and a plane-wave basis set with a kinetic energy cutoff extended to 500 eV was taken for all calculations. The core electrons are described with the Projector Augmented Wave (PAW) method (*7, 8*). A special quasi-random structure (SQS) model with 324 atoms of $RbAg_4I_5$ was generated by Alloy Theoretic Automated Toolkit (ATAT) code(*9*)to lead to a reasonable periodic supercell approximation for the partially occupied structure. The energy convergence criteria of all calculations were set to 10-4 eV.

PDF method: The synchrotron x-ray total scattering measurements were carried out at the BL02B1 beamline at SPring-8 using the rapid acquisition PDF method (RAPDF)(*10-12*). The $RbAg_4I_5$ powder sample was loaded in a 1-mm-diameter polymide capillaries and measured at room temperature. The experimental setup was calibrated by measuring the crystalline Si as a standard

material. A two-dimensional large area flat panel detector was mounted behind the sample perpendicular to the primary beam path with a sample-to-detector distance of 129.9872 mm. The incident x-ray wavelength was 0.2480 Å.

The detector exposure time was 5 s for the sample, and the number of frames taken for the sample was adjusted to be 10 for sufficient counting statistics on the data, so the total exposure time was 50 s. The collected data frames were summed, corrected for polarization effects, and masked to remove outlier pixels before being integrated along arcs of constant Q, where $Q = 4\pi sin(\theta)/\lambda$ is the magnitude transfer on scattering, to produce 1D powder diffraction patterns using the pyFAI program (*13*) Standardized corrections and normalizations were then applied to the data to obtain the total scattering structure function, *F(Q)*, which was Fourier transformed to obtain the PDF using PDFgetX3(*14*) The minimum and maximum range of data used in the Fourier transform was chosen to be $Q_{min} = 0.1\ \text{Å}^{-1}$ and $Q_{max} = 14.5\ \text{Å}^{-1}$, so as to give the best trade-off between statistical noise and real-space resolution.

Thermal conductivity model: Considering there are four different types of Ag atoms, which generate 60 lattice sites by symmetry that are partially occupied by Ag, the generated the special quasi-random structure (SQS) model is too big for the phonon calculation. The thermal conductivity of single-crystalline $RbAg_4I_5$ is predicted based on three assumptions: i) the phonon dispersions of acoustic and optical phonons are sinusoidal and Einstein-mode (*15*); ii) Umklapp-process scattering (U) and point defect scattering (PD) are taken into account for acoustic phonon scattering, while the optical ones are treated with minimum relaxation times (*16*); iii) the point defect scattering derives from the partial occupancy of Ag_1, Ag_2 and Ag_3, which is averaged to be 0.25 based on the refinement in Table S2. The mass fluctuation is estimated from the mass difference between the fully occupied and vacant Ag, while the strain fluctuation is estimated from the bond length fluctuation according to the X-ray atomic pair distribution functions (PDF) analyses (Figure S4). The parameters used for the modelling are: average atomic mass of 1.91×10^{-25} kg, average atomic volume of 3.55×10^{-29} m^3, average sound speed of 1071 m s^{-1}, Grüneisen parameter of 1.64*(1.4-0.001**T*) where *T* is the absolute temperature, mass fluctuation is determined to be 0.026 and strain fluctuation is 0.005, and number of atoms in the primitive cell is 40. From the peak position and full width at half maximum of Ag-I bonds according to the PDF results, the disordered Ag^+ ions induce high mean square strain of ~0.5% (Figure S4). Consequently, $RbAg_4I_5$ shows the lowest κ among dense solids including the skeleton materials of porous heat insulators(*17, 18*) (Figure S5). It is worth noting that the introduction of assumptions regarding no point defect scattering and average sound velocity under similar atomic masses 1786 m/s is hypothetically used to illustrate the complex partially occupied structure of $RbAg_4I_5$ and the important influence of the decrease in sound velocity caused by heavy elements on the reduction of thermal conductivity (Figure 1f).

The κ-model of porous materials consists of the contributions from solid (κ_s) and gas (κ_g) phases (*19, 20*). To describe the geometry of the pores, two variables of pore wall thickness (*D*) and side length of the cavity (*d*) are introduced by approximating the porous structure as hollow cubic boxes. The relation between relative density (ρ), wall thickness and side length can be,

$$\rho = \frac{\rho^*}{\rho^0} = 1 - \frac{V_{\text{porous}}}{V_{\text{all}}} = 1 - \frac{Nd^3}{\big((N+1)D + Nd\big)^3} \approx 1 - \frac{d^3}{(D+d)^3} \qquad \text{S1}$$

Where V_{porous} is the total volume of cavities, and V_{all} is the total volume of the porous material. At any given ρ, the side length of the cavity (*d*) in the skeleton can be determined using the wall thickness (*D*). The *D* and *d* are determined under the following boundary conditions: 1) the minimal *d* is estimated to be 3.34 nm according to the equivalent side length of free air (22.4 liter per molar gas at 300 K under a standard atmospheric pressure), because an smaller *d* would require extra pressure to squeeze gas molecules entering the pore; and 2) the minimal *D* is set to be 1 nm, because the lattice parameter of most inorganics are smaller than 1 nm.

A model for the contribution of interfacial thermal resistance to the total solid-phase thermal resistance of aerogels was constructed based on Matthiessen's rule(*21*, *28*)):

$$\frac{1}{\Lambda_0} = \frac{1}{\Lambda_{\text{v}}} + \frac{1}{\Lambda_{\text{s}}} + \frac{1}{\Lambda_{\text{T}}} \qquad \text{S2}$$

where Λ_{v}, Λ_{s} and Λ_{T} represent the contributions of intrinsic phonon scattering, boundary scattering, and interfacial scattering to the effective mean free path, respectively. Λ_{v} can be estimated from the parameters of the bulk material(*22*):

$$\Lambda_{\text{v}} = \Lambda_{\text{dense skeleton}} = \frac{3\kappa_{\text{dense skeleton}}}{C_{\text{v}} v_{\text{dense skeleton}}} \qquad \text{S3}$$

If all the particles in the aerogel backbone are assumed spherical, then interfacial scattering can be calculated by (*22*):

Supplementary

$$\Lambda_{\rm T} = \frac{3sa^2}{4d_{\rm p}^2} \quad \text{S4}$$

where s represents the center-to-center distance of adjacent particles, a is the contact diameter, and $d_{\rm p}$ is the particle diameter. $\Lambda_{\rm s}$ can be calculated with the boundary area weighted average

$$\frac{1}{\Lambda_{\rm s}} = \frac{1}{a}\frac{2A_{\rm inter}}{A_{\rm eff}} + \frac{1}{d_{\rm p}}\frac{A_{\rm sphere}}{A_{\rm eff}} \quad \text{S5}$$

where $A_{\rm inter}$ is the contact area with $A_{\rm inter} = \pi a^2/4$ and $A_{\rm sphere}$ is the area of spherical cap with $A_{\rm sphere} = \pi d_{\rm p} s$, $A_{\rm eff}$ is the effective scattering boundary area of a single particle with $A_{\rm eff} = A_{\rm inter} + 2A_{\rm sphere}$.The results when the ratio of contact diameter to solid particle diameter is 0.5 are shown in Figure S12.

At high relative densities (e.g. when the particle size is greater than 100 nanometers), the interfacial effects and size effects can be neglected. As the density decreases (e.g. when the aerogel particle size is less than 10 nanometers), the intrinsic phonon scattering also accounts for 85% of the overall thermal conductivity. However, in low-density model fitting, the proportion of the skeleton gradually decreases, and the error range gradually narrows. To avoid additional errors introduced by the model when considering the interfacial thermal resistance, the total κ of porous materials assume a heat flux in parallel of $\kappa = \kappa_{\rm s} + \kappa_{\rm g}$ (*23, 24*)

$\kappa_{\rm s}$ is estimated according to the phonon diffusion model(*26*) taking into accounts of both reductions in relative density (ρ) and mean sound velocity (v)

$$\kappa_{\rm s} = \kappa_{\rm s}^0 \rho \frac{v}{v_0} \quad \text{S6}$$

The correction factor (c) between v and ρ (*27*) is obtained to be 0.6 according an exponential fitting (*7*) to measurements (Figure S9).

$$\frac{v}{v_0} = \rho^c \quad \text{S7}$$

As the density decreases, the proportion of the skeleton gradually decreases, the error range gradually reduces. So ultimately ignoring the interfacial thermal resistance, setting the bulk thermal conductivity ($\kappa_{\rm s}^0$) at 125 mW/m-K, the corresponding values of $\kappa_{\rm s}$ for porous materials can be calculated by substituting the porosity into the above equations.

$\kappa_{\rm g}$ is estimated according to Kaganer (*29*) taking into account of Kundsen number(*30*) of reductions in thermal conductivity and flowing speed due to viscosity at the solid-gas interface, once the pore size is smaller than the mean free path of 74 nm for free air at 300 K under a standard atmospheric pressure.

$$\kappa_{\rm g} = \kappa_{\rm g}^0 \frac{1}{1 + 2\beta Kn} = \kappa_{\rm g}^0 \frac{1-\rho}{1 + 2\beta \frac{l_0}{d}} \quad \text{S8}$$

The constant β is defined as

$$\beta = \frac{5\pi}{32}\frac{(2-\alpha)}{\alpha}\frac{(9\gamma - 5)}{\gamma + 1} \quad \text{S9}$$

Among the parameter variables, $\kappa_{\rm g}^0$ is 26 mW/m-K, mean free path of free gas (l_0) is 74 nm, α of 1 is the thermal adaptation coefficient and γ of 1.4 is the air adiabatic coefficient (*31*). By substituting the relative density ρ and the values of d derived from different assumed values of D, the corresponding distinct values of $\kappa_{\rm g}$ can be obtained.

Supplementary

Table R1. A list of parameters used in the thermal conductivity fitting model.

Parameter	
Relative Density	ρ (%)
Density	ρ^* (cm^3/g)
Pore wall thickness	D (nm)
Side length of the cavity	d (nm)
Thermal conductivity of encapsulated gas in the pores	κ_g (mW/m-K)
Thermal conductivity of solid forming skeleton	κ_s (mW/m-K)
Kundsen number	Kn
sound velocity	v (m/s)
Correction factor of sound velocity in aerogel	c
Mean free path of free gas	l_0 (nm)

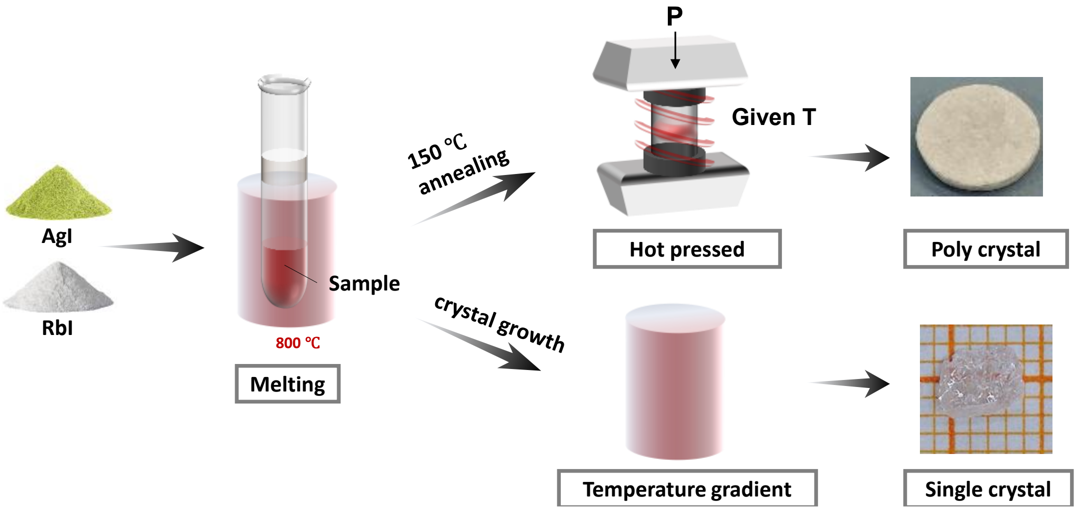

Figure S1. Schematic for the growth of $RbAg_4I_5$.

Supplementary

Table S2. Crystallographic data obtained from single-crystal X-ray diffraction refinements at 300 K.

Formula	$RbAg_4I_5$
Molecular weight, g·mol^{-1}	1151.18
Crystal size, mm^3	0.4 × 0.4 × 0.3
Crystal system	cubic
Space group	$P4_132$
T, K	300
$a=b=c$, Å	11.2467(2)
$\alpha=\beta=\gamma$, °	90
V, Å^3	1422.58(8)
Z	4
D_{calcd}, g/cm^3	5.375
F(000)	1960
μ, mm^{-1}	19.620
θ range, °	2.56-29.11
reflections collected	18074
independent reflections (R_{int})	763 (0.0601)
reflections observed [$I > 2\sigma(I)$]	679
data/restraints/parameters	763/1/36
R_1, wR_2 ($I > 2\sigma(I)$)	0.0446, 0.1310
R_1, wR_2 (all data)	0.0486, 0.1355
GooF on F^2	1.129
$\Delta\rho_{max}$, $\Delta\rho_{min}$, e·Å^{-3}	0.884/-1.452
position $x/y/z$ of Rb	0.375000/0.375000/0.375000
position $x/y/z$ of Ag_1	0.5288(4)/0.2686(5)/0.7953(4)
position $x/y/z$ of Ag_2	0.9927(6)/0.8520(10)/0.2107(6)
position $x/y/z$ of Ag_3	0.1722(11)/0.1722(11)/0.1722(11)
Site occupancy of Ag_1	0.367
Site occupancy of Ag_2	0.255
Site occupancy of Ag_3	0.133
position $x/y/z$ of I_1	0.03010(7)/0.03010(7)/0.03010(7)
position $x/y/z$ of I_2	0.375000/0.82231(1)/0.92768(9)
mean-square displacements $U_{11}/U_{22}/U_{33}/U_{23}/U_{13}/U_{12}$ of Rb (Å^2)	0.1053(16)/0.1053(16)/0.1053(16)/-0.0315(12)/-0.0315(12)/-0.0315(12)
mean-square displacements $U_{11}/U_{22}/U_{33}/U_{23}/U_{13}/U_{12}$ of Ag_1 (Å^2)	0.083(2)/0.120(3)/0.102(3)/0.002(2)/0.0144(19)/0.003(2)
mean-square displacements $U_{11}/U_{22}/U_{33}/U_{23}/U_{13}/U_{12}$ of Ag_2 (Å^2)	0.071(3)/0.177(8)/0.119(6)/0.004(5)/-0.003(3)/0.011(4)
mean-square displacements $U_{11}/U_{22}/U_{33}/U_{23}/U_{13}/U_{12}$ of Ag_3 (Å^2)	0.132(12)/0.132(12)/0.132(12)/-0.036(7)/-0.036(7)/-0.036(7)
mean-square displacements $U_{11}/U_{22}/U_{33}/U_{23}/U_{13}/U_{12}$ of I_1 (Å^2)	0.0599(4)/0.0599(4)/0.0599(4)/-0.0010(3)/-0.0010(3)/-0.0010(3)
mean-square displacements $U_{11}/U_{22}/U_{33}/U_{23}/U_{13}/U_{12}$ of I_2 (Å^2)	0.0666(7)/0.0658(5)/0.0658(5)/-0.0041(5)/-0.0020(4)/-0.0020(4)

Supplementary

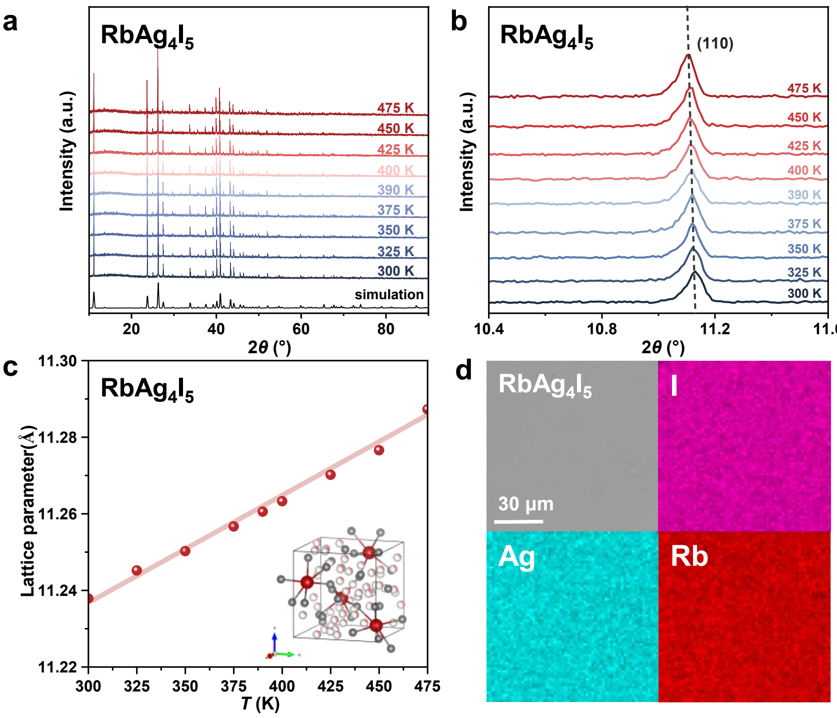

Figure S2. Temperature dependent X-ray powder diffraction patterns (a, b) for $RbAg_4I_5$. Temperature dependent lattice parameters showing a positive thermal expansion coefficient along the crystallographic a/b/c-axis directions (c). SEM image and corresponding EDS mappings of a typical cleaved single crystalline surface (d).

Table S3. Measured transverse (v_t), longitudinal (v_l) and average (v) sound velocities, and estimated Debye temperature (θ_D), shear modules (G), bulk modulus (B), Grüneisen parameter (γ) and linear thermal expansion coefficient (α) for single crystalline $RbAg_4I_5$ at 300 K.

Formula	SC-$RbAg_4I_5$
v_t	962
v_l	1733
v	1071
θ_D	97
G (GPa)	5.0
B (GPa)	9.5
γ	1.6
α ($\times 10^{-6}$ K^{-1})	25

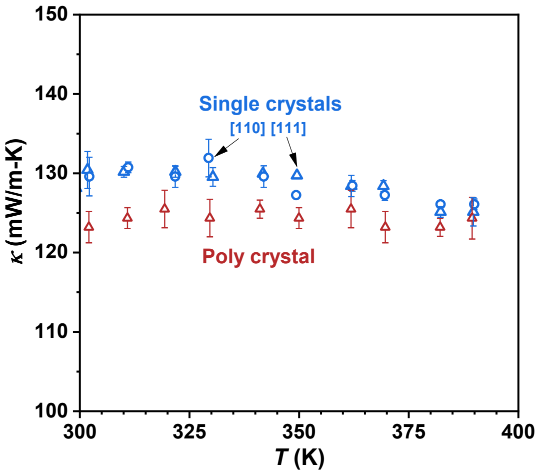

Figure S3. Thermal conductivity within 300~400 K for single- and poly-crystalline $RbAg_4I_5$.

Supplementary

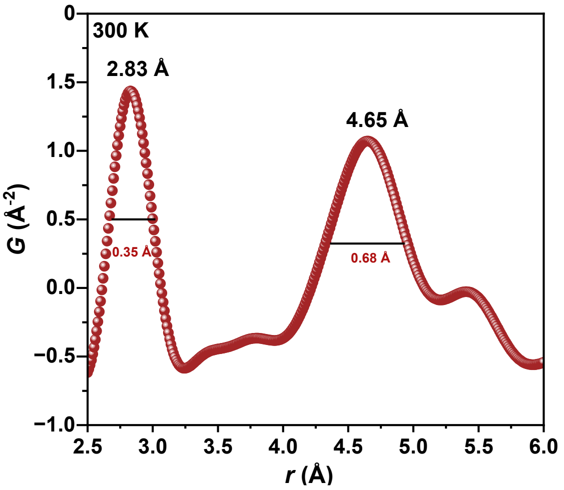

Figure S4. Room-temperature X-ray atomic pair distribution function (PDF) datasets for polycrystalline $RbAg_4I_5$.

Table S4. Mean sound velocities of materials having similar average atomic mass as compared to that of $RbAg_4I_5$.

Compounds	Average mass (g/mol)	Mean sound velocity (m/s)	Reference
RbI	106.19	1318	Sirdeshmukh(*32*)
CsBr	106.40	1549	Sirdeshmukh(*32*)
$AgGaTe_2$	108.20	1894	Chen(*16*)
$CuInTe_2$	108.39	2002	Chen(*16*)
Sm_3Se_4	109.56	2119	Tamaki(*33*)
$CuSbTe_2$	110.13	2085	Chen(*16*)
β-HgS	116.33	2054	Kumazaki(*34*)
CdSb	117.09	1846	Madelung(*35*)
InSb	118.29	2188	Drabble(*36*)
$AgBiSe_2$	118.69	1620	Chen(*16*)
$TlInSe_2$	119.28	1506	Chen(*16*)
$AgInTe_2$	119.47	1673	Chen(*16*)
PbS	119.63	2128	Wang(*37*)
TlCl	119.92	1352	Kodama(*38*)
CdTe	120.01	1651	Bijalwan(*39*)
$AgSbTe_2$	121.21	1662	Chen(*16*)
$AgIn_5Te_8$	121.63	1787	Chen(*16*)
$NaBiTe_2$	121.79	1871	Chen(*16*)
2H-CdI_2	122.07	1350	Sandercock(*40*)
SnTe(Li)	123.16	2064	Li(*41*)

Supplementary

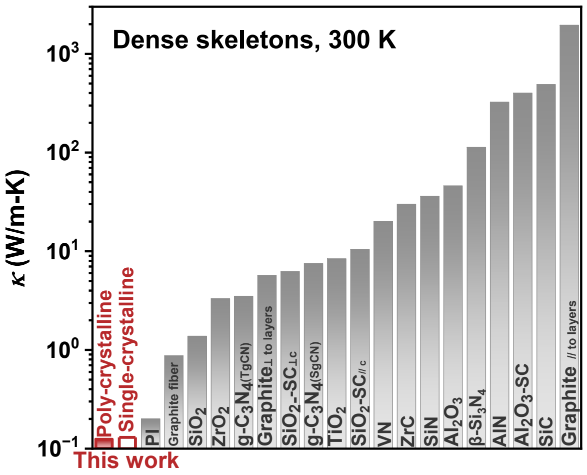

Figure S5. Room-temperature thermal conductivity for single-crystalline and polycrystalline $RbAg_4I_5$, as compared to that of existing skeletons of porous heat insulators (more details and the relevant references are given in Table S5).

Table S5. Room-temperature thermal conductivity for the skeletons in dense form of existing porous heat insulators.

Compounds	Thermal conductivity (W/m-K)	Reference
Polyimide	0.2	Touloukian(*17*)
Graphite fiberepoxy(25% vol) ⊥ to fibers	0.87	Touloukian(*17*)
SiO_2(poly crystals)	1.38	Touloukian(*17*)
ZrO_2	3.3	Hostaša(*42*)
g-C_3N_4(TgCN)	3.5	Mortazavi(*18*)
Graphite(pyrolytic κ ⊥ to layers)	5.7	Touloukian(*17*)
SiO_2(single crystals ⊥ to c axis)	6.21	Touloukian(*17*)
g-C_3N_4(SgCN)	7.5	Mortazavi(*18*)
TiO_2 (poly crystal)	8.4	Touloukian(*17*)
SiO_2(single crystals//to c axis)	10.4	Touloukian(*17*)
VN	20	Zheng(*43*)
ZrC	30	Tiwari(*44*)
SiN	36	Touloukian(*17*)
Al_2O_3 (poly crystal)	46	Touloukian(*17*)
β-Si_3N_4	113	Zhou(*45*)
AlN	325	Touloukian(*17*)
Al_2O_3 (single crystal)	400	Touloukian(*17*)
SiC	490	Touloukian(*17*)
Graphite (pyrolytic κ// to layers)	1950	Touloukian(*17*)

Supplementary

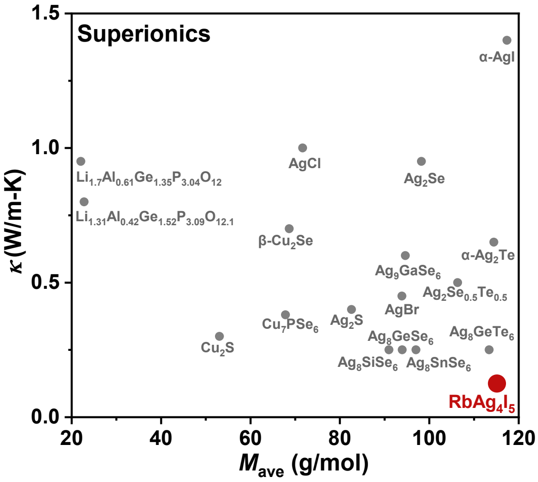

Figure S6. Room-temperature thermal conductivity of ionic conductors as a function of average atomic mass (M_{ave}) (*46-54*).

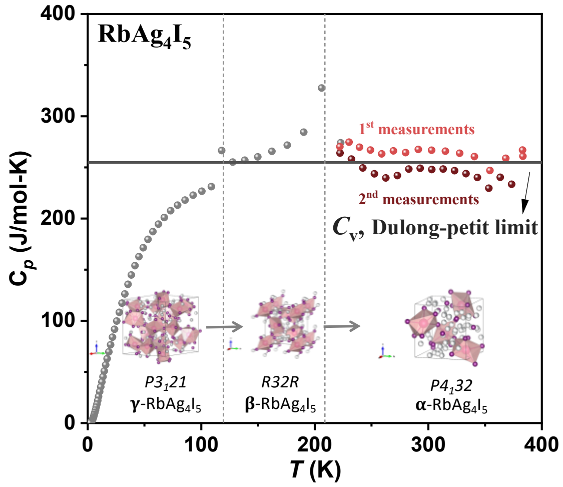

Figure S7. Temperature dependent specific heat for single-crystalline $RbAg_4I_5$, as compared to the estimation of Dulong-petit limit.

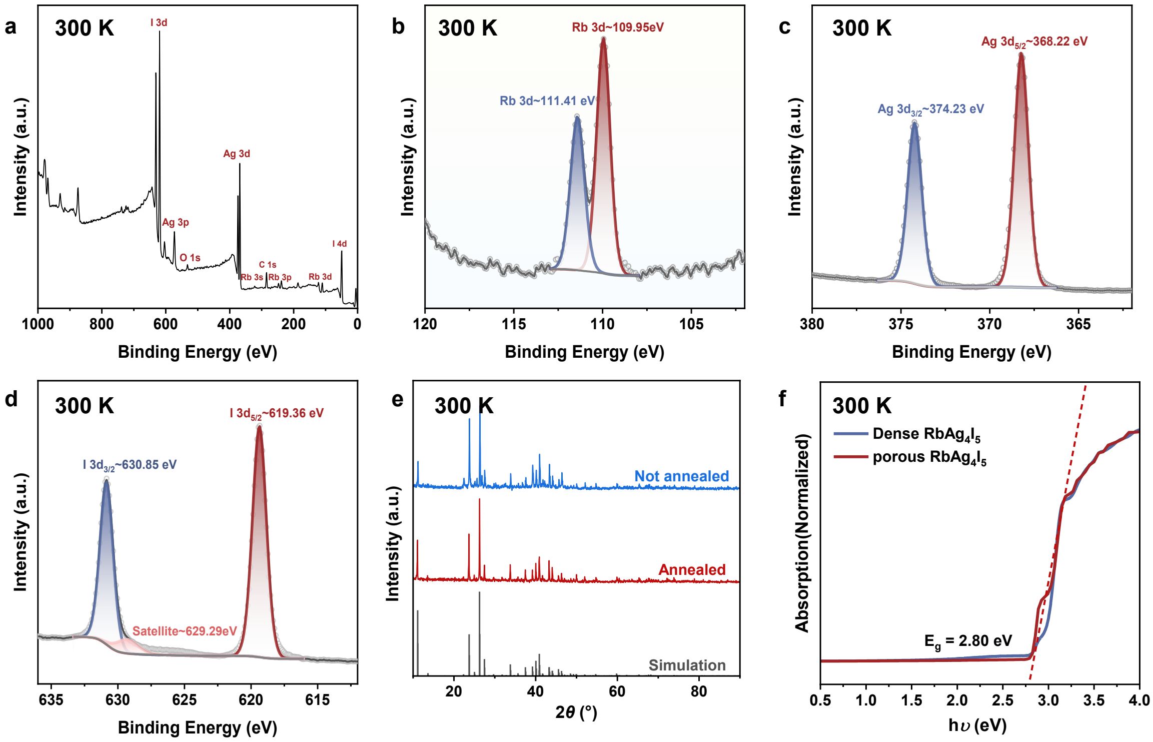

Figure S8. Characterization of polycrystalline porous materials using X-ray photoelectron spectroscopy (a-d) for $RbAg_4I_5$, XRD patterns for solution-processed $RbAg_4I_5$ (e) and optical absorption measurements for $RbAg_4I_5$ synthesized by melting and by solution-based techniques.

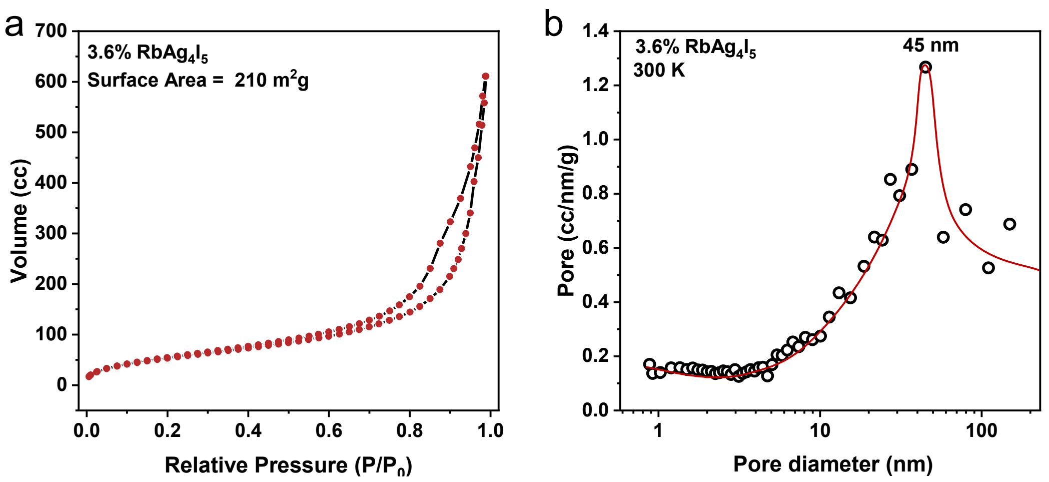

Figure S9. (a) N_2-aspirated attachment curves for the 3.6% dense $RbAg_4I_5$ sample and the corresponding distribution of pore diameter (b).

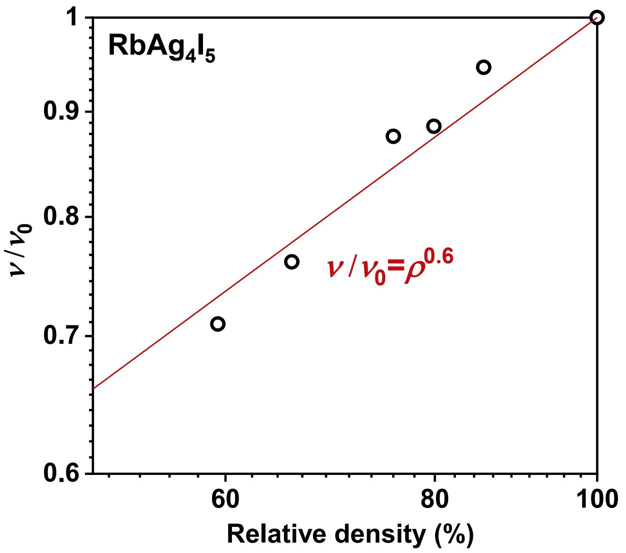

Figure S10. Room temperature sound velocity as a function of density for $RbAg_4I_5$ with exponential fitting.

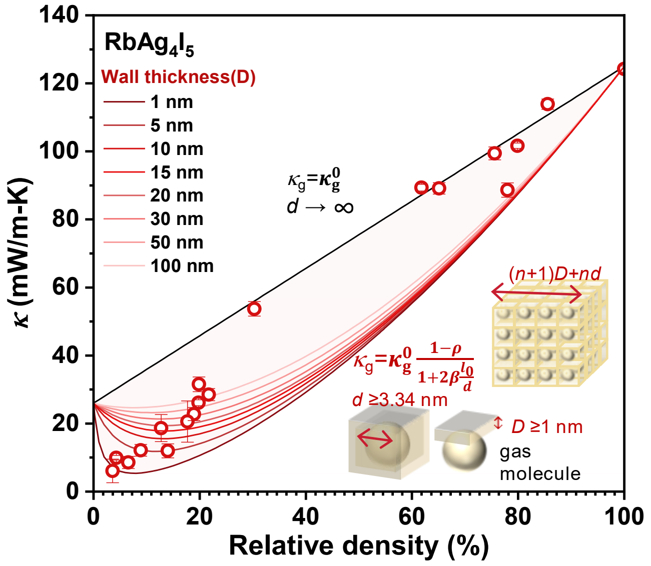

Figure S11. Density dependent room-temperature thermal conductivity for $RbAg_4I_5$, which a comparison to model predictions based on the schematic structure (lower panels) with different wall thicknesses (D) and side length (d) of uniformly distributed cubic pores.

Supplementary

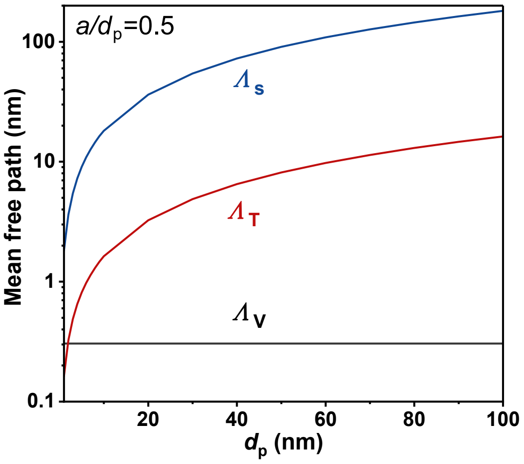

Figure S12. Contributions of various thermal resistances in $RbAg_4I_5$. d_p is the particle size, and a is the particle contact diameter. Λ_V, Λ_S and Λ_T represent the mean free path due to the intrinsic phonon scattering, boundary scattering and interface scattering, respectively.

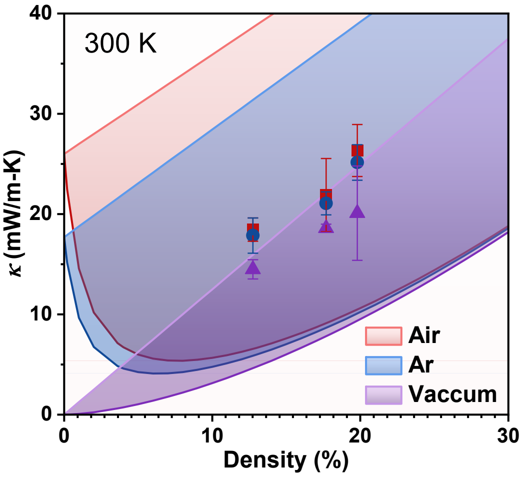

Figure S13. A comparison between measurements and model predictions for porous $RbAg_4I_5$ under different atmospheres.

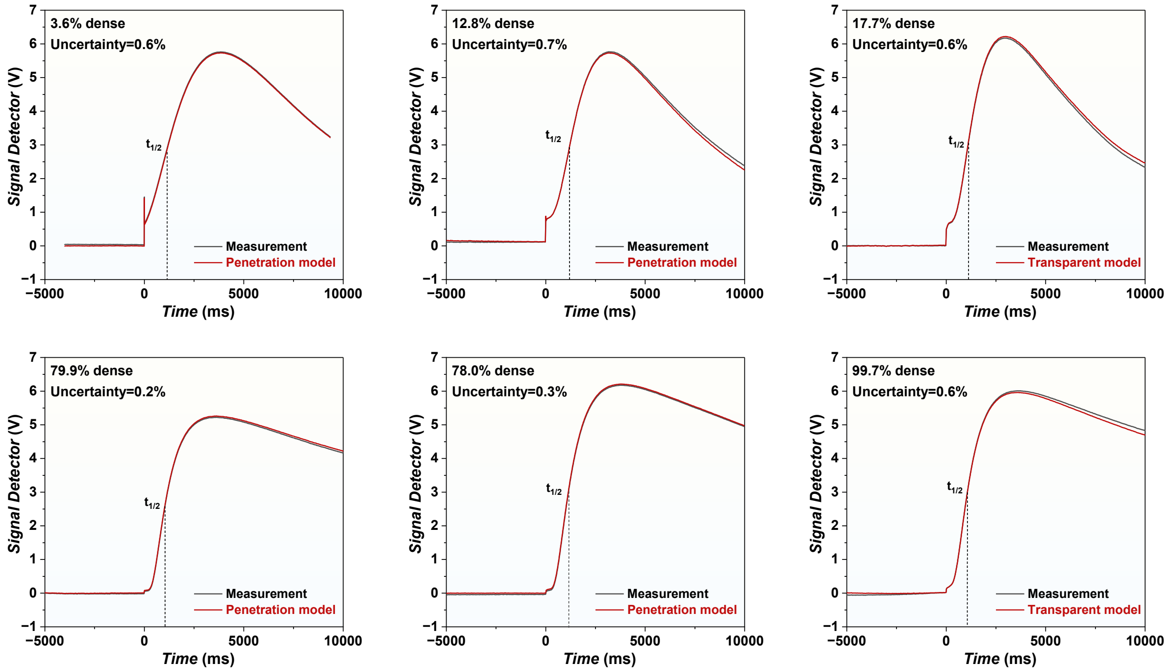

Figure S14. Raw data of thermal diffusivity measurement at 300 K via laser flash method, for a series of porous samples.

Supplementary

Table S6. Solubility test results of measured sample mass in 200mL water

Sample	Weight at 0 day	Weight at 3 days
blank	0.6577 g	0.6556 g
1	1.0231 g	0.7518 g
2	1.6182 g	1.3503 g

Table S7. Room-temperature thermal conductivity for porous materials from the literatures.

Materials	Thermal conductivity (mW/m-K)	Reference
SiC@SiO_2	14	Su(*55*)
Pure SiO_2 aerogel	15.9	Zhao(*56*)
SiO_2/chitosan aerogels	17.8	Meti(*57*)
Zeolitic imidazolate aerogels	18	Bendahou(*58*)
Liquid-crystalline nanocellulose aerogels（LC-NCell aerogels）	18	Kobayashi(*59*)
Graphene aerogels	19	Zu(*60*)
Hexagonal boron nitride aerogels	20	Xu(*61*)
Cellulose /SiO_2 aerogels	21	Ahankari(*62*)
Amorphous silica aerogels	22.1	Huang(*63*)
SiO_2 aerogel/fiber skeleton composites	23	An(*64*)
Polyimide/bacterial cellulose aerogels	23	Zhang(*65*)
Bleached cellulose fibers aerogels （BCF based aerogels）	23	Seantier(*66*)
Carbon-SiO_2-Al_2O_3 aerogels	24	Lv(*67*)
Bleached cellulose fibers/cellulose nanocrystals (BCF/ NFC composite aerogels)	25	Seantier(*66*)
Graphene amorphous boron nitride aerogels (a-BNGA aerogels)	25.41	Yu(*68*)
Zircon nanofibrous aerogels	26	Guo(*69*)

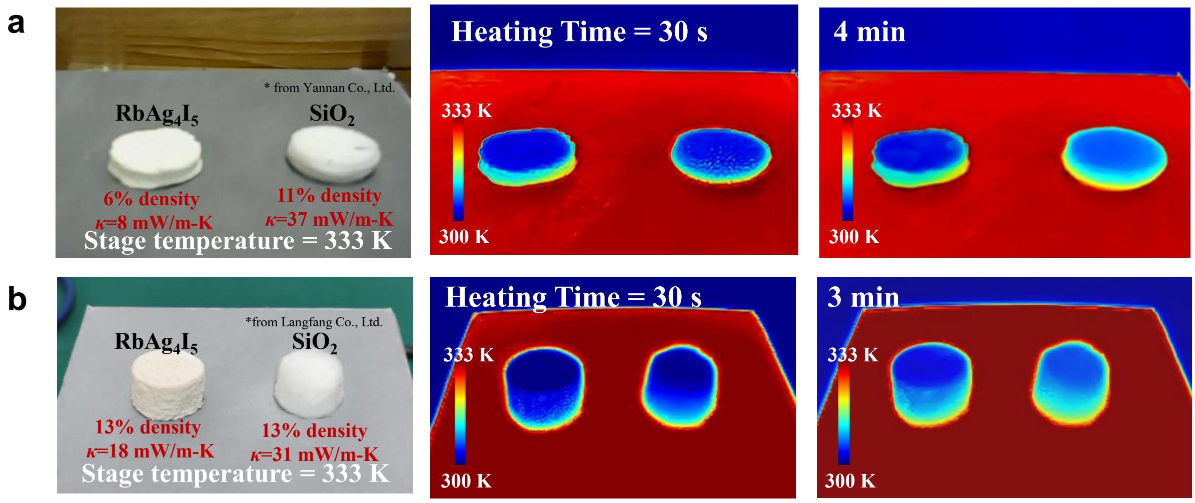

Figure S15. Time dependent distribution of temperature for $RbAg_4I_5$ and commercial SiO_2 aerogel with different densities/thermal conductivities placed on a hot stage of 333 K monitored by both optical and infrared cameras.

Supplementary

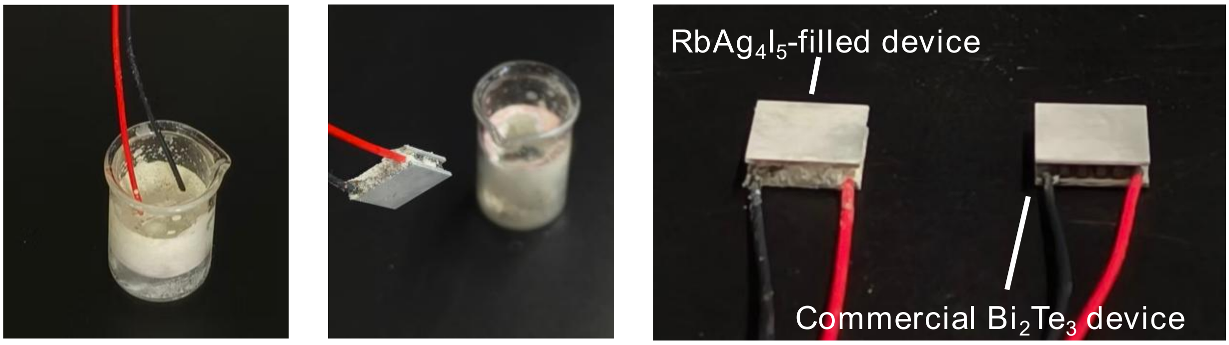

Figure S16. Typical photos of the thermoelectric devices with or without the $RbAg_4I_5$ filling.

Table S8. Quality inspection table of commercial Bi_2Te_3 thermoelectric device (Model 9501/017/030 B from Ferrotec).

Type	TE9501/017/030B/T
Length and width	11.5±0.50 mm
Hight	3.18±0.025 mm
Resistance	0.48-0.58 Ω (25 °C)

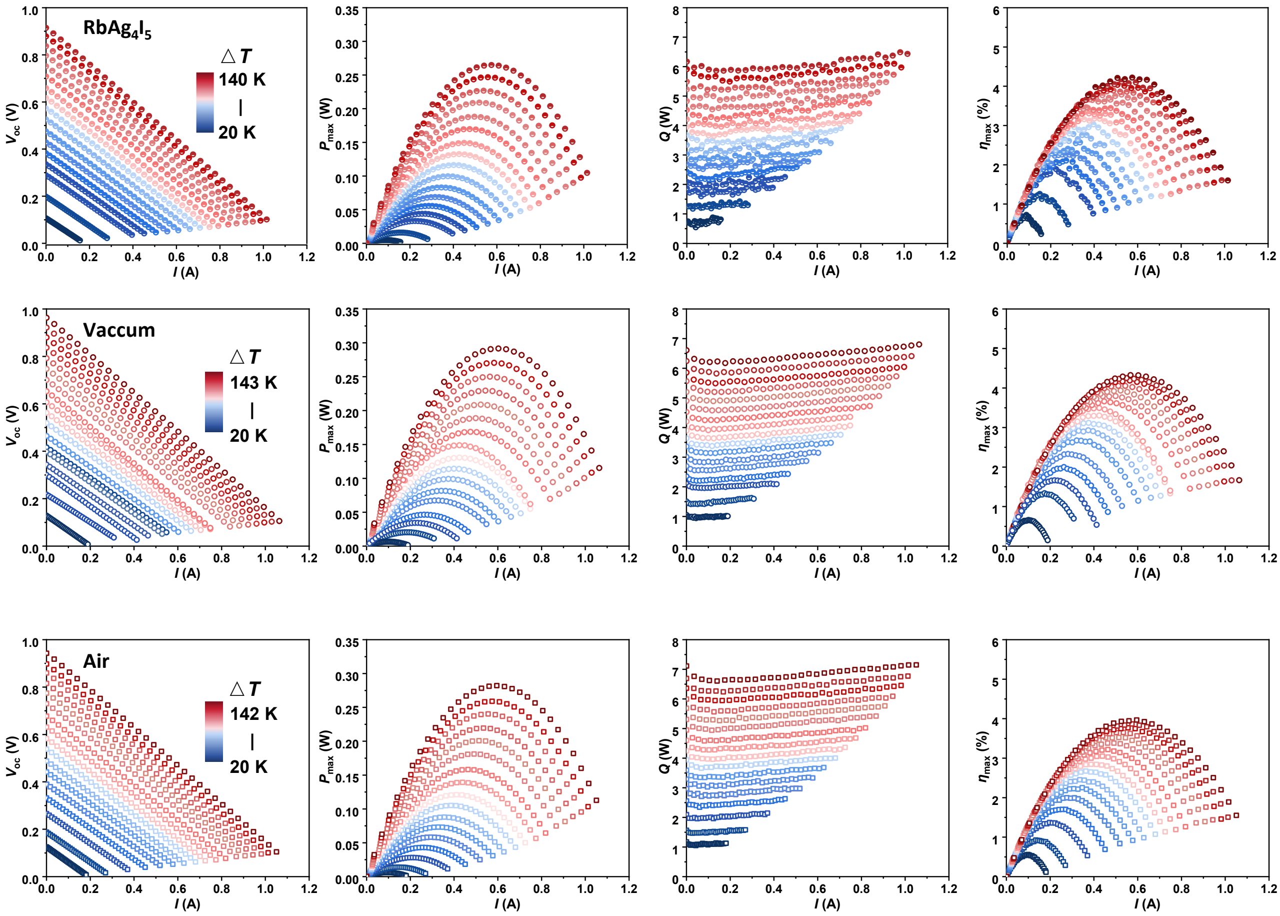

Figure S17. Power generation performance. (a) Open-circuit voltage (V_{OC})(b) maximum output power (P_{max}), (c) heat flow (Q), and (d) conversion efficiency (η_{max}) as a function of different temperature gradients (ΔT) for commercial Bi_2Te_3 device and 3.6% dense $RbAg_4I_5$-filled device.

Supplementary

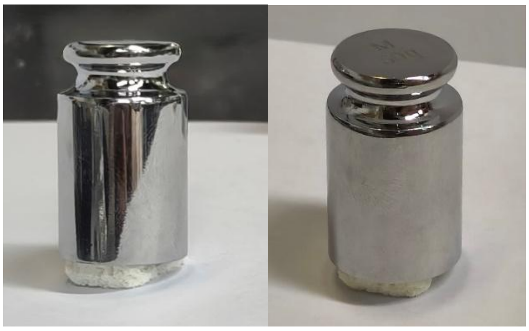

Figure S18. The load-bearing capacity of the sample used for the CPU thermal insulation with a weight ratio of 1:208.

Supplementary

Supplementary references

1. O. V. Dolomanov, L. J. Bourhis, R. J. Gildea, J. A. Howard, H. Puschmann, OLEX2: a complete structure solution, refinement and analysis program. *Journal of applied crystallography* **42**, 339-341 (2009).
2. G. M. Sheldrick, SHELXT–Integrated space-group and crystal-structure determination. *Acta Crystallographica Section A: Foundations and Advances* **71**, 3-8 (2015).
3. T. B. a. A. Ono, Improvement of the laser flash method to reduce uncertainty in thermal diffusivity measurements. *Meas. Sci. Technol.* **12**, 2046-2057 (2001).
4. G. Kresse, J. Hafner, Ab initiomolecular-dynamics simulation of the liquid-metal–amorphous-semiconductor transition in germanium. *Physical Review B* **49**, 14251-14269 (1994).
5. J. F. G. Kresse, Efficient iterative schemes for ab initio total-energy calculations using a plane-wave basis set. *Physical Review B* **54**, 11169-11186 (1996).
6. K. B. J.P. Perdew, M. Ernzerhof, Generalized Gradient Approximation Made Simple. *Physical Review B* **77**, 3865-3868 (1996).
7. P. E. Blöchl, Projector augmented-wave method. *Physical Review B* **50**, 17953-17979 (1994).
8. D. J. G. Kresse, From ultrasoft pseudopotentials to the projector augmented-wave method. *Physical Review B* **59**, 1758-1775 (1999).
9. A. Van De Walle, Asta, M., and Ceder, G, The alloy theoretic automated toolkit: A user guide. *Calphad* **26**, 539-553 (2002).
10. P. J. Chupas, Qiu, X., Hanson, J. C., Lee, P. L., Grey, C. P., Billinge, S. J. L, Rapid-Acquisition Pair Distribution Function (RA-PDF) Analysis. *J Appl Cryst* **36**, 1342–1347 (2003).
11. T. Egami, Billinge, S. J. L., in *Pergamon materials series,* E. Amsterdam, Ed. (2012).
12. S. Billinge *et al.*, in *Comprehensive Inorganic Chemistry III*. (2021), pp. 222–247.
13. J. Kieffer, D. Karkoulis, PyFAI, a versatile library for azimuthal regrouping. *Journal of Physics: Conference Series* **425**, (2013).
14. P. D. Juhás, T.; Farrow, C. L.; Billinge, S. J. L, PDFgetX3: A Rapid and Highly Automatable Program for Processing Powder Diffraction Data into Total Scattering Pair Distribution Functions. *J Appl Cryst* **46**, 560–566 (2013).
15. R. Xu *et al.*, Realizing Plain Optimization of the Thermoelectric Properties in BiCuSeO Oxide via Self-Substitution-Induced Lattice Dislocations. *Research* **6**, 0123 (2023).
16. Z. Chen, X. Zhang, S. Lin, L. Chen, Y. Pei, Rationalizing phonon dispersion for lattice thermal conductivity of solids. *National Science Review* **5**, 888-894 (2018).
17. Y. S. Touloukian, Powell, R. W., Ho, C. Y. & Klemens, P. G. , *Thermal conductivity*. Nonmetallic solids (Springer Us, UK, 1970).
18. B. Mortazavi, G. Cuniberti, T. Rabczuk, Mechanical properties and thermal conductivity of graphitic carbon nitride: A molecular dynamics study. *Computational Materials Science* **99**, 285-289 (2015).
19. J. Fan *et al.*, Ultralow thermal conductivity in graphene–silica porous ceramics with a special saucer structure of graphene aerogels. *Journal of Materials Chemistry A* **7**, 1574-1584 (2019).
20. M. Yang *et al.*, Dual role of two-dimensional graphene in silica aerogel composite: Thermal resistance and heat node. *Colloids and Surfaces A: Physicochemical and Engineering Aspects* **699**, (2024).
21. C. D. G. Chen, Theoretical phonon thermal conductivity of Si/Ge superlattice nanowires. *Journal of Appplied Physics* **95**, 682-693 (2003).
22. J. D. Jackson, *Classical Electrodynamics*. 3rd Edition (John Wiley & Sons, Inc., New York, 1998).
23. P. G. Collishaw, J. R. G. Evans, An assessment of expressions for the apparent thermal conductivity of cellular materials. *Journal of Materials Science* **29**, 2261-2273 (1994).
24. E. Solórzano, M. A. Rodriguez-Perez, J. Lázaro, J. A. de Saja, Influence of solid phase conductivity and cellular structure on the heat transfer mechanisms of cellular materials: diverse case studies. *Advanced Engineering Materials* **11**, 818-824 (2009).
25. L. J. Gibson, M. F. Ashby, *Cellular solids : structure and properties*. (Cellular solids : structure and properties, 1997).
26. J. Fricke *et al.*, in *Insulation Materials, Testing and Applications*. (ASTM International, 1990), vol. STP1030-EB, pp. 0.
27. G. H. Tang, C. Bi, Y. Zhao, W. Q. Tao, Thermal transport in nano-porous insulation of aerogel: Factors, models and outlook. *Energy* **90**, 701-721 (2015).
28. G. Tang, C. Bi, Y. Zhao, W. Tao, Thermal transport in nano-porous insulation of aerogel: Factors, models and outlook. *Energy* **90**, 701-721 (2015).
29. M. G. Kaganer, Thermal insulation in cryogenic engineering. *Israel Program for Scientific Translations, Jerusalem*, (1969).
30. D. M. Ruthven, in *Encyclopedia of Physical Science and Technology (Third Edition),* R. A. Meyers, Ed. (Academic Press, New York, 2003), pp. 251-271.
31. F. Hemberger, S. Weis, G. Reichenauer, H.-P. Ebert, Thermal transport properties of functionally graded carbon aerogels. *International Journal of Thermophysics* **30**, 1357-1371 (2009).
32. D. B. Sirdeshmukh, L. Sirdeshmukh, K. Subhadra, *Alkali Halides: A Handbook of physical properties*. (Springer Science & Business Media, 2013), vol. 49.
33. A. Tamaki *et al.*, Valence fluctuation of Sm_3Se_4. *Journal of Physics C: Solid State Physics* **18**, 5849 (1985).
34. K. Kumazaki, Elastic properties and ionicity of zero-gap semiconductors. *physica status solidi (a)* **33**, 615-623 (1976).
35. O. Madelung, *Semiconductors: data handbook*. (Springer Science & Business Media, 2004).
36. J. Drabble, A. Brammer, The third-order elastic constants of indium antimonide. *Proceedings of the Physical Society* **91**, 959 (1967).
37. H. Wang, E. Schechtel, Y. Pei, G. J. Snyder, High thermoelectric efficiency of n-type PbS. *Advanced Energy Materials* **3**, 488-495 (2013).
38. M. Kodama, S. Saito, S. Minomura, Pressure Dependence of the Elastic Constants of TlCl. *Journal of the Physical*

Society of Japan **33**, 1361-1371 (1972).
39. R. Bijalwan, P. Ram, M. Tiwari, Lattice thermal conductivity of II-VI compounds. *Journal of Physics C: Solid State Physics* **16**, 2537 (1983).
40. J. Sandercock, Some recent developments in Brillouin scattering. *Rca Rev* **36**, 89-107 (1975).
41. W. Li *et al.*, Band and scattering tuning for high performance thermoelectric $Sn_{1-x}Mn_xTe$ alloys. *Journal of Materiomics* **1**, 307-315 (2015).
42. J. Hostaša, W. Pabst, J. Matějíček, Thermal conductivity of Al_2O_3–ZrO_2 composite ceramics. *Journal of the American Ceramic Society* **94**, 4404-4409 (2011).
43. Q. Zheng *et al.*, Phonon and electron contributions to the thermal conductivity of VN_x epitaxial layers. *Physical Review Materials* **1**, 065002 (2017).
44. J. Tiwari, T. Feng, Intrinsic thermal conductivity of ZrC from low to ultrahigh temperatures: A critical revisit. *Physical Review Materials* **7**, 065001 (2023).
45. H. Zhou, T. Feng, Theoretical upper limits of the thermal conductivity of Si_3N_4. *Applied Physics Letters* **122**, (2023).
46. H. Liu *et al.*, Copper ion liquid-like thermoelectrics. *Nature Materials* **11**, 422-425 (2012).
47. F. Drymiotis, T. W. Day, D. R. Brown, N. A. Heinz, G. Jeffrey Snyder, Enhanced thermoelectric performance in the very low thermal conductivity $Ag_2Se_{0.5}Te_{0.5}$. *Applied Physics Letters* **103**, (2013).
48. Y. He *et al.*, Ultrahigh Thermoelectric Performance in Mosaic Crystals. *Advanced Materials* **27**, 3639-3644 (2015).
49. W. Li *et al.*, Low Sound Velocity Contributing to the High Thermoelectric Performance of Ag_8SnSe_6. *Advanced Science* **3**, (2016).
50. F. Tesfaye, M. Moroz, An Overview of Advanced Chalcogenide Thermoelectric Materials and Their Applications. *Journal of Electronic Research and Application* **1**, 13 (2018).
51. T. Wang, H.-Y. Chen, P.-F. Qiu, X. Shi, L.-D. Chen, Thermoelectric properties of Ag_2S superionic conductor with intrinsically low lattice thermal conductivity. *Acta Physica Sinica* **68**, (2019).
52. T. Bernges *et al.*, Considering the Role of Ion Transport in Diffuson-Dominated Thermal Conductivity. *Advanced Energy Materials* **12**, (2022).
53. T. R. Wei, P. Qiu, K. Zhao, X. Shi, L. Chen, Ag_2Q-Based (Q = S, Se, Te) Silver Chalcogenide Thermoelectric Materials. *Advanced Materials* **35**, (2022).
54. A. Ghata *et al.*, Exploring the Thermal and Ionic Transport of Cu^+ Conducting Argyrodite Cu_7PSe_6. *Advanced Energy Materials* **14**, (2024).
55. L. Su *et al.*, Anisotropic and hierarchical SiC@ SiO_2 nanowire aerogel with exceptional stiffness and stability for thermal superinsulation. *Science advances* **6**, eaay6689 (2020).
56. S. Zhao *et al.*, Additive manufacturing of silica aerogels. *Nature* **584**, 387-392 (2020).
57. P. Meti *et al.*, Overview of organic–inorganic hybrid silica aerogels: Progress and perspectives. *Materials & Design* **222**, 111091 (2022).
58. D. Bendahou, A. Bendahou, B. Seantier, Y. Grohens, H. Kaddami, Nano-fibrillated cellulose-zeolites based new hybrid composites aerogels with super thermal insulating properties. *Industrial Crops and Products* **65**, 374-382 (2015).
59. Y. Kobayashi, T. Saito, A. Isogai, Aerogels with 3D ordered nanofiber skeletons of liquid-crystalline nanocellulose derivatives as tough and transparent insulators. *Angewandte Chemie International Edition* **53**, 10394-10397 (2014).
60. G. Zu *et al.*, Superelastic multifunctional aminosilane-crosslinked graphene aerogels for high thermal insulation, three-component separation, and strain/pressure-sensing arrays. *ACS applied materials & interfaces* **11**, 43533-43542 (2019).
61. X. Xu *et al.*, Double-negative-index ceramic aerogels for thermal superinsulation. *Science* **363**, 723-727 (2019).
62. S. Ahankari, P. Paliwal, A. Subhedar, H. Kargarzadeh, Recent developments in nanocellulose-based aerogels in thermal applications: a review. *ACS nano* **15**, 3849-3874 (2021).
63. D. Huang, M. Zhang, L. Shi, Q. Yuan, S. Wang, Effects of particle size of silica aerogel on its nano-porous structure and thermal behaviors under both ambient and high temperatures. *Journal of Nanoparticle Research* **20**, 1-15 (2018).
64. Z. An, X. Hou, P. Zhou, R. Zhang, D. Fang, A novel flexible, layered, recoverable SiO_2 fiber skeleton and aerogel composites material prepared by papermaking process. *Ceramics International* **47**, 12963-12969 (2021).
65. X. Zhang *et al.*, Bidirectional anisotropic polyimide/bacterial cellulose aerogels by freeze-drying for super-thermal insulation. *Chemical Engineering Journal* **385**, 123963 (2020).
66. B. Seantier, D. Bendahou, A. Bendahou, Y. Grohens, H. Kaddami, Multi-scale cellulose based new bio-aerogel composites with thermal super-insulating and tunable mechanical properties. *Carbohydrate Polymers* **138**, 335-348 (2016).
67. Y. Lv *et al.*, Design of the Thermal Restructured Carbon–Inorganic Composite Aerogel for Efficient Thermal Protection of Aero-Engines. *ACS Applied Materials & Interfaces* **14**, 38185-38195 (2022).
68. H. Yu *et al.*, Chemically bonded multi-nanolayer inorganic aerogel with a record-low thermal conductivity in a vacuum. *National Science Review* **10**, nwad129 (2023).
69. J. Guo *et al.*, Hypocrystalline ceramic aerogels for thermal insulation at extreme conditions. *Nature* **606**, 909-916 (2022).
